# Cachexia in chronic obstructive pulmonary disease: new insights and therapeutic perspective

**DOI:** 10.1002/jcsm.12062

**Published:** 2015-09-07

**Authors:** Karin J. C. Sanders, Anita E. M. Kneppers, Coby van de Bool, Ramon C. J. Langen, Annemie M. W. J. Schols

**Affiliations:** ^1^ Department of Respiratory MedicineNUTRIM School of Nutrition and Translational Research in MetabolismMaastrichtThe Netherlands

**Keywords:** COPD, Muscle, Emphysema, Energy balance, Cachexia, Lung cancer

## Abstract

Cachexia and muscle wasting are well recognized as common and partly reversible features of chronic obstructive pulmonary disease (COPD), adversely affecting disease progression and prognosis. This argues for integration of weight and muscle maintenance in patient care. In this review, recent insights are presented in the diagnosis of muscle wasting in COPD, the pathophysiology of muscle wasting, and putative mechanisms involved in a disturbed energy balance as cachexia driver. We discuss the therapeutic implications of these new insights for optimizing and personalizing management of COPD‐induced cachexia.

**List of abbreviations**
p70S6K: ribosomal protein S6 kinase, 70kDa, polypeptide 1 (PRPS6KB1)
S6: ribosomal protein S6 (RPS6)
4EBP1: eukaryotic translation initiation factor 4E binding protein 1 (EIF4EBP1)
AEE: activity induced energy expenditure
AKT: signalling through v‐akt murine thymoma viral oncogene
AMPK: AMP‐activated protein kinase
ASM: appendicular skeletal muscle mass
ATROGIN1: F‐box protein 32 (FBXO32)
BAT: brown adipose tissue
BMD: bone mineral density
BMI: body mass index
BNIP3: BCL2/ adenovirus E1B 19kDa interacting protein 3
COPD: chronic obstructive chronic disease
CT: computed tomography
DALY: disability‐adjusted life years
DEXA: dual‐energy X‐ray absorptiometry
DIT: diet‐induced energy expenditure
FFM: fat‐free mass
FFMI: fat‐free mass index
FM: fat mass
FOXO: forkhead box O transcription factor
HIF1‐α: hypoxia inducible factor 1, alpha subunit
IGF1: insulin‐like growth factor 1
LC3: microtubule‐associated protein 1 light chain 3 alpha (MAP1LC3A)
MSTN: myostatin
MtDNA: mitochondrial DNA
MTORC1: mechanistic target of rapamycin (serine/threonine kinase) complex 1
MURF1: tripartite motif containing 63, E3 ubiquitin protein ligase (TRIM63)
MYOD1: myogenic differentiation 1
MYOG: myogenin
NEDD4: neural precursor cell expressed, developmentally downregulated 4, E3 ubiquitin protein
NF‐κB: nuclear factor kappa‐light‐chain‐enhancer of activated B cells
NSCLC: non‐small cell lung cancer
OXPHEN: loss of muscle oxidative phenotype
P62: sequestosome 1 (SQSTM1)
PI3K: phosphatidylinositol‐4,5‐bisphosphate 3‐kinase, catalytic subunit alpha (PIK3CA)
PMA: pectoralis muscle area
PPARG: peroxisome proliferator‐activated receptor gamma
REE: resting energy expenditure
TEE: total energy expenditure
TUNEL: terminal deoxynucleotidyl transferase
UCP: uncoupling proteins
ULK1: unc‐51 like autophagy activating kinase 1
UPS: ubiquitin proteasome system
VEGF: endothelial growth factor
WAT: white adipose tissue

## Introduction

Chronic obstructive pulmonary disease (COPD) is one of the leading causes of death worldwide. It has been estimated that COPD‐related mortality rates will even increase in the coming decades. This increase is not only related to the prevalence of smoking, but also ageing and reduced mortality from other common causes of death play a role.^1^ Additionally, COPD is a major contributor to global disease burden, accounting for 43.3 million disability‐adjusted life years in 2010.^2^ The disease is characterized by persistent airflow obstruction, resulting from inflammation and remodelling of the airways, and may include development of emphysema. Furthermore, systemic disease manifestations and acute exacerbations influence disease burden and mortality risk.^3^ Extending the classical descriptions of the ‘pink puffer’ and ‘blue bloater’, recent unbiased statistical approaches^4^, ^5^ support the concept that body weight and body composition discriminate pulmonary phenotypes and are predictors of outcome. Extra‐pulmonary degenerative manifestations that may occur in COPD include osteoporosis^6^ and muscle wasting. The prevalence of muscle wasting is relatively high in COPD: 15–40% depending on definition and disease stage.^7^, ^8^ Importantly, muscle wasting not only contributes to diminished skeletal muscle function, reduced exercise capacity, and decreased health status,^9^, ^10^ but is also a determinant of mortality in COPD, independent of airflow obstruction.^8^, ^11^

Muscle wasting in COPD has been demonstrated by decreases in fat‐free mass (FFM) at whole body level, but also specifically at the level of the extremities.^12^ Whole body and trunk FFM reduction are more pronounced in the emphysematous phenotype, whereas reduced FFM in extremities is not different between the pulmonary phenotypes.^13^, ^14^ In addition, muscle wasting is apparent as a decrease in the size of individual muscle fibres, and this muscle fibre atrophy in COPD seems selective for type II fibres in peripheral muscle,^15^, ^16^ which is in line with other chronic diseases prone to cachexia such as chronic heart failure.^17^ Furthermore, a shift in muscle fibre composition from type I (oxidative) to type II (glycolytic), accompanied by a decrease in oxidative capacity, culminates in reduced muscle endurance.^18^ This not only contributes to reduced exercise capacity^19^ but may also affect muscle mass in COPD,^20^ because type I and II fibres display different responses to anabolic and catabolic signals.^21^, ^22^

While unintended weight loss was initially considered to be an indicator of inevitable and terminal progression of the disease process, there is now convincing evidence that it is an independent determinant of survival, arguing for weight maintenance in patient care. There are indications that the pathophysiology of unintended weight loss is different between clinically stable COPD and during acute flare‐ups of the disease. To date, data in acute exacerbations of COPD are, however, very limited. Therefore, lung cancer is used as a comparative acute pulmonary cachexia model.

A recent unbiased statistical approach suggests that not all COPD patients but only the emphysematous phenotype is prone to cachexia,^4^ although the informative value of available clinical studies is limited by a cross‐sectional study design. The last two decades have also yielded insight in the impairments of the processes governing muscle mass and identified putative triggers of muscle wasting in COPD. However, it remains unclear to what extent acute flare‐ups of COPD may accelerate chronic wasting of muscle mass and whether muscle wasting involves similar mechanisms as in other chronic diseases or in lung cancer cachexia. In this review, we present recent insights in the pathophysiology of muscle wasting in COPD and (putative) mechanisms involved in the pathophysiology of a disturbed energy balance as important driver of cachexia, which may lead to novel targets for clinical management of cachexia in COPD.

## Recent developments in identifying muscle wasting in COPD

Incorporation of body composition into nutritional assessment has been a major step forward in understanding systemic COPD pathophysiology, since changes in weight and classification of body mass index (BMI) do not account for (hidden) body compositional shifts in fat mass (FM), FFM, and bone mineral density. In clinical research, bioelectrical impedance analysis is commonly used to identify cachexia. Traditionally, reference values for fat‐free mass index (FFMI) in COPD were developed based on age‐specific and gender‐specific 10th percentile values.^8^ These reference values were defined as abnormally low, based on well‐established adverse effects of low FFMI on physical performance and survival in normal to underweight COPD patients.^7^, ^11^ However, this might underestimate low muscle mass in the increasing proportion of overweight to obese COPD patients.^23^ The recent European Respiratory Society statement on nutritional assessment and therapy in COPD^24^ proposed dual‐energy X‐ray absorptiometry (DEXA) as the most appropriate method for body composition analysis in COPD, mostly because it combines screening for osteoporosis with assessment of FM and FFM at the regional level in addition to whole body level. Consequently, body composition assessed by DEXA also allows measurement of appendicular skeletal muscle mass (ASM), which has been demonstrated to be stronger related to physical functioning than total FFM.^23^ Moreover, we recently showed that this particularly applies to overweight to obese COPD patients.^23^, ^25^

Whereas low muscle mass is prevalent in ±15% of well‐functioning elderly in the general aged population,^26^ a higher prevalence can be expected in COPD as a reflection of accelerated ageing.^25^ Indeed, Van de Bool *et al*. recently identified low ASM in even 87% of Dutch COPD patients eligible for pulmonary rehabilitation, along with a high persisting prevalence across all BMI categories.^23^ The ASM‐wasted phenotype was not only associated with impaired strength but in men also with decreased endurance capacity. Coexistent abdominal obesity was identified in 78% of muscle‐wasted patients, which appeared to have a protective effect on physical functioning.^23^ Physiological alterations in terms of less hyperinflation and a larger inspiratory capacity in obese COPD patients contribute to certain advantages during physical activity.^27^ In addition, mortality rates in advanced COPD are the lowest among obese subjects.^28^ This prognostic advantage typically reflects the ‘obesity paradox’, because obesity, on the other hand, is also associated with increased risk of cardiovascular and metabolic diseases.

Although clinically useful estimates can be derived by DEXA, a more precise distinction between muscle mass, visceral and subcutaneous adipose tissue requires more advanced imaging technologies. This could be relevant in COPD as Van den Borst *et al*. and Furutate *et al.* recently reported a higher visceral adipose tissue in older‐age patients with COPD compared with age‐matched healthy controls, despite comparable subcutaneous adipose tissue and BMI.^29^, ^30^

McDonald *et al*.^31^ recently demonstrated that CT‐derived pectoralis muscle area (PMA) provides a more clinically relevant measure of COPD‐related outcomes in comparison with BMI, as lower PMA was associated with more severe expiratory airflow obstruction, lower quality of life, and impaired exercise capacity. Because gender differences have been documented in body composition and its functional implications,^23^ Diaz *et al*. explored gender differences in computed tomography (CT)‐derived PMA and observed lower PMA in women compared with men.^32^ CT scans are often used to exclude other underlying illnesses, and therefore, chest CT‐derived analysis of body composition may be an attractive diagnostic tool to combine screening for pulmonary and systemic pathology. However, it first needs to be properly validated against reference methods of whole body and regional body composition to allow use in clinical practice. Furthermore, due to the radiation exposure, use of CT scans for body composition assessment is only admissible when scans are already performed to screen for pulmonary pathology.

## New insights in the pathophysiology of muscle wasting in chronic obstructive pulmonary disease

The loss of muscle mass and cross sectional area in COPD patients as determined by imaging techniques has been confirmed at the cellular level, that is, a reduction in muscle fibre cross‐sectional area.^16^ As reviewed by Langen *et al.* 33 and Remels *et al.*,^20^ triggers of muscle wasting include hypoxemia, oxidative stress, inflammation, impaired growth factor signalling, oral glucocorticoids, disuse, and malnutrition, some of which are influenced by smoking.^34^ Wasting of skeletal muscle is due to a net catabolic state, which may result from an imbalance in muscle protein synthesis and breakdown (protein turnover), as well as from an imbalance in myonuclear accretion and loss (myonuclear turnover).

### Protein turnover

To get insight in the rate of (muscle) protein turnover in COPD, information on (muscle) protein synthesis and breakdown is required. Both increased and normal rates of whole body protein turnover have been reported in patients with COPD,^35^, ^36^ but the relative contribution of muscle versus other tissues to protein turnover is unknown. Rutten *et al*. observed an increase in myofibrillar protein breakdown in cachectic COPD patients compared with non‐cachectic patients and controls,^35^ but no data are available regarding muscle protein synthesis rate, except for a small study showing depressed muscle protein synthesis rates in malnourished patients with emphysema.^37^ Numerous studies, however, have addressed molecular regulation of anabolic and catabolic pathways in the quadriceps muscle of COPD patients, which provides some insight in altered muscle protein turnover in muscle‐wasted COPD patients.

### Proteolytic signalling

Several environmental triggers can lead to catabolic signalling in the skeletal muscle, mediated by transcriptional regulators including nuclear factor kappa‐light‐chain‐enhancer of activated B cells (NF‐κB) and forkhead box O transcription factors (FOXOs). NF‐κB activity is increased in COPD patients compared with controls^38^, ^39^ and in cachectic COPD patients compared with non‐cachectic COPD patients.^38^, ^40^ Furthermore, limb muscle NF‐κB activity is increased in patients with lung cancer cachexia.^41^ FOXO mRNA and protein expression are increased in patients with COPD,^38^, ^39^, ^42^, ^43^, ^44^, ^45^ seemingly independent of body composition, although it is noticeable that in all studies, the patient group showed signs of emphysema. In the COPD patient group, FOXO‐1 protein expression was higher in limb muscles than in respiratory muscles, while this difference was not found in controls.^46^ Interestingly, the respiratory muscles of COPD patients show an opposite fibre type shift compared with limb muscles, that is, towards more type I fibres.^47^, ^48^ This will have implications for the expression levels of constituents of atrophy signalling pathways.^22^, ^49^ Increased catabolic signalling through FOXO and NF‐κB can induce gene expression of key factors in both the ubiquitin proteasome system (UPS)^50^, ^51^ and the autophagy lysosome pathway.^33^, ^52^

### Ubiquitin proteasome‐mediated degradation

The ubiquitin 26S‐proteasome pathway consists of coordinated actions of the ubiquitin conjugating and ligating enzymes that link ubiquitin chains onto proteins to mark them for degradation by the proteasome.^53^, ^54^ These enzymes include tripartite motif containing 63, E3 ubiquitin protein ligase (TRIM63, referred to as MURF1), F‐box protein 32 (FBXO32, referred to as ATROGIN1), and neural precursor cell expressed, developmentally downregulated 4, E3 ubiquitin protein (NEDD4).

MURF1 limb and respiratory muscle mRNA and protein expression appear unaltered in COPD patients compared with controls,^38^, ^40^, ^55^, ^56^, ^57^ although one study reported increased MURF1 protein expression in the limb muscles of cachectic COPD patients.^39^ In COPD patients, MURF1 protein expression is relatively increased in limb muscle than in the respiratory muscle, while this difference was not found in controls.^46^ Furthermore, cachectic COPD patients show increased limb muscle mRNA expression of MURF1 compared with a control population.^44^ ATROGIN1 mRNA and protein expression are increased in limb muscles,^38^, ^39^, ^40^, ^44^, ^56^, ^57^ but unaltered in respiratory muscles of patients with COPD.^55^ Similarly, ATROGIN1 mRNA expression is increased in the limb muscles of smokers.^58^ Furthermore, Doucet *et al*. found that COPD patients display a higher ATROGIN1 protein expression in limb muscles than in respiratory muscles, while this difference was not found in controls.^46^ Additionally, limb muscle NEDD4 protein expression is increased in patients with COPD.^57^ Total poly‐ubiquitinated protein is increased in limb muscles of COPD patients compared with healthy controls^39^, ^56^ and in cachectic COPD patients compared with non‐cachectic COPD patients.^38^

Taken together, the majority of the literature suggests that wasting in COPD is accompanied by an increase in UPS activation. The increase in catabolic signalling in cachectic COPD patients is site specific. This may reflect disuse atrophy of the limb muscle with maintained or increased respiratory muscle activity, or it may result from an interaction between inactivity and other triggers of atrophy**,** such as smoking.

### Autophagy‐lysosome‐mediated degradation

The autophagy‐lysosome pathway is a protein degradation pathway, which recently gained interest in the context of COPD‐associated muscle dysfunction. Upon activation, autophagosomes form and mature to subsequently fuse with lysosomes. The autophago‐lysosomes degrade the cargo and release amino‐acids for *de novo* protein synthesis or other metabolic fates.^59^

Signalling through v‐akt murine thymoma viral oncogene (AKT) regulates mechanistic target of rapamycin (serine/threonine kinase) complex 1 (MTORC1) activity and downstream of MTORC1, unc‐51 like autophagy activating kinase 1 (ULK1) activity, thereby regulating autophagy initiation.^60^, ^61^ Inhibitory MTORC1‐mediated ULK1 phosphorylation is decreased in limb muscles of COPD patients compared with controls,^45^ which may implicate an increase in autophagic flux induction. The increase of FOXO mRNA and protein expression in COPD patients may induce the transcription of autophagy‐related genes. However, it should be taken into account that FOXO transcriptional activity is also regulated by post‐translational modifications. Plant *et al*. found that the mRNA expression of autophagy‐related genes Beclin‐1 and microtubule‐associated protein 1 light chain 3 alpha (MAP1LC3A, referred to as LC3) is unaltered in the limb muscles of COPD patients compared with controls.^57^ Limb muscle sequestosome 1 (SQSTM1, referred to as P62) mRNA expression, however, is increased in COPD patients.^45^ Although the mRNA expression of autophagy‐related genes and the activation of ULK1 may give some insight in the level of autophagy initiation, it does not directly reflect the level of autophagic flux. In muscle biopsies, the conversion of LC3BI to LC3BII can be used as a measure for autophagic flux. Furthermore, P62 is used as a marker for autophagic flux, because it is broken down by the lysosome. In the limb muscles of COPD patients, the LC3BII/I ratio is increased,^39^, ^45^ pointing to an increase in the autophagic flux. In contrast, P62 protein expression is increased, pointing to a decrease in autophagic flux.^45^ However, it cannot be excluded that the increase in P62 protein expression is due to the increase in P62 transcription. The number of autophagosomes was found to be increased in the limb muscle of COPD patients,^39^, ^45^ which suggests an increase in autophagic flux. However, it is only possible to speculate on the level of autophagic flux in the limb muscles of patients with COPD based on the currently applied markers, as these incompletely cover autophagic flux, autophagy induction, and autophagic‐lysosomal degradation. Therefore, autophagic flux markers should be analysed coupled to the activity status of upstream regulators such as MTORC1 and AMP‐activated protein kinase (AMPK) in muscle biopsies. Moreover, besides its role in protein breakdown, autophagy also acts as a quality control mechanism for proteins and intracellular components.^62^, ^63^ Therefore, an impaired autophagic flux in COPD patients may have consequences for the integrity and function of intracellular components such as the nucleus and mitochondria.

It currently is unknown if the autophagic‐lysosome pathway activity is altered during acute exacerbations of COPD, because most studies were conducted in stable COPD patients. However, in lung cancer cachexia, LC3BII protein expression and BCL2/ adenovirus E1B 19kDa interacting protein 3 (BNIP3) mRNA expression are induced,^41^ pointing to an increase in autophagy. From this, autophagy induction in skeletal muscle might be anticipated during acute stages of COPD wasting.

### Protein synthesis signalling

A major anabolic pathway is the insulin‐like growth factor 1 (IGF1)/phosphatidylinositol‐4,5‐bisphosphate 3‐kinase, catalytic subunit alpha (PIK3CA referred to as PI3K)/AKT pathway. Most studies found an increase in IGF1 mRNA expression in the limb muscle of COPD patients compared with controls,^42^, ^43^, ^64^ although Crul *et al*. found a decrease in IGF1 mRNA expression in stable COPD patients.^65^ Unfortunately, this study did not provide body composition data. Cachectic COPD patients seem to have a lower limb muscle IGF1 mRNA and protein expression than non‐cachectic COPD patients.^40^ Furthermore, during an acute exacerbation, muscle IGF1 mRNA expression is lower in COPD patients than in controls, although IGF1 protein expression remains unaltered.^65^

Even though IGF1 mRNA expression is increased in limb muscles of COPD patients, AKT activation remains unaltered.^39^, ^45^, ^57^ AKT activity is relatively increased in cachectic patients compared with non‐cachectic patients and healthy controls,^40^, ^44^ while the decrease in IGF1 mRNA expression in this group would generally implicate a decrease of IGF1/AKT signalling. Interestingly, an increase in AKT activation is also observed in patients with lung cancer‐related cachexia,^41^ suggesting it may be a common feature of pulmonary cachexia. The discrepancy in IGF1 mRNA expression and AKT activation suggests altered regulation at the IGF‐receptor or IGF‐receptor protein expression level.^66^

Signalling through AKT inhibits the upstream inhibitor of MTORC1, thereby inducing MTORC1 activation and subsequent phosphorylation of its downstream targets eukaryotic translation initiation factor 4E‐binding protein 1 (EIF4EBP1, also called 4EBP1) and ribosomal protein S6 kinase, 70kDa, polypeptide 1 (PRPS6KB1, also called p70S6K).^67^ The increased AKT activation in the limb muscle of cachectic patients compared with non‐cachectic COPD patients is paralleled by an increase in phosphorylation of the downstream targets 4E‐BP1 and p70S6K.^44^ P70S6K phosphorylation is unaltered in COPD patients compared with controls,^44^ while ribosomal protein S6 (RPS6 referred to as S6) phosphorylation was even decreased in COPD patients.^45^ Together, these studies show an increase in protein synthesis signalling in the limb muscles of cachectic COPD patients compared with non‐cachectic COPD patients, but no alteration in the general COPD population. In patients with lung cancer‐related cachexia, AKT activation is increased without concurrent activation of MTOR or its downstream targets.^41^ This may indicate that, although impaired AKT signalling is found in lung cancer cachexia, AKT signalling is largely intact in COPD‐induced muscle wasting. However, one limitation of these studies concerns the evaluation of the activation status of protein synthesis signalling solely in a basal state. Although the current data may suggest that the protein synthesis pathway is a promising target for the treatment of COPD‐induced muscle wasting, the integrity of the anabolic response should be further addressed.

Only limited data are available on anabolic signalling in respiratory muscles of COPD patients, and although the results also point to an increase in anabolic signalling, it remains unclear if this is different between cachectic and non‐cachectic COPD patients. Martinez‐Llorens *et al*. found an increase in IGF1 mRNA expression in the intercostal muscles of patients with COPD.^68^ Doucet *et al*. compared the ratio of quadriceps to diaphragm AKT activation in COPD patients with controls and found a lower ratio in COPD.^20^ This implicates that the AKT activation is relatively higher in the diaphragm than in the quadriceps. In line with this, the p70S6K phosphorylation is relatively higher in the diaphragm, while 4E‐BP1 phosphorylation is higher in the quadriceps.^20^

Interestingly, Tannerstedt *et al*. showed a difference in anabolic response between type‐I and type‐II muscle fibres, with increased AKT phosphorylation and downstream pathway activation in the type‐II fibres.^69^ In contrast, in COPD, the respiratory muscle with a shift towards more type‐I fibres displayed a larger AKT activation and downstream signalling than the limb muscles with a shift towards more type‐II fibres. Therefore, the shift in fibre type does not explain the variation in AKT phosphorylation and downstream signalling between the limb muscle and respiratory muscle. Other discriminating factors, such as the muscle activity level, may be more closely linked to differences in AKT phosphorylation.

Taken together, anabolic signalling is increased in the skeletal muscle of patients with COPD, with an even larger increase in the diaphragm than the limb muscles. One may speculate that the increased activation of AKT signalling in the respiratory muscles is an attempt to preserve respiratory function by compensating catabolic triggers, although it may also reflect intrinsic alterations in muscle fibre composition.

### Myonuclear turnover

Besides the turnover of proteins, the turnover of myonuclei appears essential for muscle regeneration.^70^, ^71^, ^72^ Furthermore, although at a lower rate, myonuclear turnover might be indispensable for the maintenance of skeletal muscle mass. The regulated loss of a nucleus may involve apoptosis. Increased apoptosis, as determined by an increase in terminal deoxynucleotidyl transferase dUTP nick end labelling (TUNEL) staining and poly (ADP‐ribose) polymerase cleavage in the skeletal muscle of cachectic COPD patients has been reported.^73^ In the diaphragm of COPD patients with muscle wasting, elevated caspase‐3 levels indicated an increase in apoptosis.^55^ No difference in apoptosis measured by TUNEL staining or caspase‐3 was found in COPD patients with maintained muscle mass compared with controls.^74^ A possible alteration in apoptosis in the muscle of COPD patients remains inconclusive due to the lack of studies and the use of different markers. Furthermore, the possible role of apoptotic signalling in the skeletal muscle atrophy remains obscure,^75^ and multinucleated muscle fibres may utilize other mechanisms for selective myonuclear loss, such as autophagy.^76^

To replenish the myonuclear pool, satellite cells are essential.^72^ In contrast to muscle wasting in ageing,^77^, ^78^ the number of satellite cells per muscle fibre is unaltered in the limb and respiratory muscles of patients with COPD compared with controls.^68^, ^79^, ^80^, ^81^ Furthermore, no differences in satellite cell number have been reported between muscle‐wasted and non‐muscle‐wasted COPD patients.^80^

Upon activation, satellite cells proliferate, differentiate, and fuse with myofibres. Activation and proliferation of satellite cells in the limb muscles does not seem to differ between COPD patients and age‐matched controls based on number of satellite cells 24 h after a resistance‐exercise bout.^81^ However, molecular markers of satellite cell activation may be more sensitive than satellite cell number to quantify the satellite cell response.

In a basal condition, myogenic factor 5 mRNA expression is unaltered in the limb muscles of patients with COPD compared with controls.^57^ Myogenic differentiation 1 (MYOD1) mRNA expression^40^ and protein expression appear similar in the limb muscles of COPD patients and healthy controls.^57^ Furthermore, Myogenin (MYOG) mRNA expression is unaltered in COPD patients.^57^ However, in cachectic COPD patients, MYOD1^40^ and MYOG^38^, ^39^ protein expression are reduced compared with controls, while no alteration in MYOG protein expression was found in the limb muscles of cachectic compared with non‐cachectic COPD patients.^38^ It should be considered that in different populations and disease stages, the course of the satellite cell response might be altered, which may have implications for the timing of the measurements.

A negative regulator of myogenesis is myostatin (MSTN).^82^ Limb muscle MSTN mRNA expression is increased in COPD compared with controls,^57^, ^83^ while no difference seems to be present between cachectic and non‐cachectic COPD patients.^40^ The increased MSTN mRNA expression in COPD patients may be partially explained by smoking status, as this increase is also found in smokers.^58^ MSTN protein expression is unaltered in the limb muscle of COPD patients, independent of the pulmonary phenotype,^38^, ^39^, ^40^ while the circulatory level of MSTN is increased in COPD compared with control subjects.^84^ It should be noted that Snijders *et al*. showed a delayed response in MSTN protein levels upon a single bout of resistance exercise in the elderly, while basal levels did not seem to differ.^85^ This implies that in the absence of a myogenic trigger, intrinsic alterations in satellite cell plasticity responses in muscle of COPD patients may be masked.

Centrally localized myonuclei in myofibres are considered derived from newly fused satellite cells prior to their final location peripherally in the myofibre against the sarcolemma. COPD patients with preserved muscle mass have higher amounts of central nuclei in the limb muscle than muscle‐wasted COPD patients and controls.^80^ This could be interpreted as an attempt to counteract atrophic signalling in COPD patients, which may be essential to preserve muscle mass. However, as there is only indirect evidence that central myonuclei reflect recent regenerative events, central nuclei could also reflect an increase in myonuclear turnover to compensate for increased loss of myonuclei.

To gain further insight in the regulation of myonuclear turnover and possible defects in COPD‐induced skeletal muscle wasting, it is essential to incorporate satellite cell activation stimuli and sensitive techniques to monitor myonuclear accretion and turnover in the study design. This will require further development of new techniques, in parallel with novel *ex vivo* and *in vitro* approaches to monitor myonuclear accretion and possibly myonuclear loss, and assessment of the role of alterations in myonuclear turnover in muscle atrophy.

### Loss of muscle oxidative phenotype

Besides the importance of the muscle quantity for muscle function, the quality of the muscle should also be considered. This is highlighted by the finding that muscle mass‐specific muscle strength and endurance are reduced in patients with COPD.^86^, ^87^, ^88^ A well‐established qualitative alteration in the skeletal muscle of COPD patients is the loss of oxidative phenotype (OXPHEN) characterized by a muscle fibre type I to type II shift and a loss of oxidative capacity.^20^, ^88^, ^89^ The loss of OXPHEN is associated with increased oxidative stress,^88^, ^90^ which may render the muscle more susceptible to muscle atrophy.^38^ In addition, type II fibres are generally more susceptible to atrophy stimuli including, for example, inflammation^21^ and hypoxia.^22^ Therefore, the loss of OXPHEN in COPD may accelerate the loss of muscle mass, thereby linking muscle quality to muscle quantity. This is supported by the fact that non‐symptomatic smokers already exhibit reduced mitochondrial capacity and a similar fibre‐type shift.^91^ Although less extensively investigated, striking similarities are reported regarding muscle oxidative metabolism in chronic heart failure.^92^ As these patients also share other systemic features and lifestyle characteristics (e.g. muscle wasting and low physical activity level) comparative analyses between well‐phenotyped patients with COPD and chronic heart failure may provide more insight in common and disease‐specific denominators and mechanisms.

### Therapeutic perspective

Because muscle wasting may result from alterations in the protein and myonuclear turnover, targeting key pathways in these processes will be required to combat muscle wasting.

Ubiquitin proteasome system activity is increased in the muscles of cachectic COPD patients, which implicates the atrogenes MURF1 and ATROGIN1 as targets to normalize UPS activity. This is supported by the finding that in a cell culture model and in a mouse model of muscle disuse, MURF1 inhibition and knockout, respectively, prevented muscle fibre atrophy.^93^, ^94^ Pharmacological inhibitors that target specific ubiquitin‐conjugating and deconjugating enzymes are being developed to treat cancer, neurodegenerative disorders, and autoimmune diseases^95^ but may also be highly relevant for the treatment of COPD‐induced muscle wasting. Furthermore, exercise training may attenuate MURF1 expression, as was observed in the skeletal muscle of chronic heart failure patients.^50^, ^96^ In contrast to exercise training, one bout of exercise leads to an increase in MURF1 expression, albeit blunted in COPD,^97^, ^98^ while the increase in proteolytic signalling is reduced by branched‐chain amino acid supplementation in a healthy population.^97^

Autophagy is disturbed in patients with COPD, although it remains unclear whether there is an increased induction of autophagy or an inhibition of autophagic‐lysosomal degradation. Low amino acid availability can activate autophagy by inhibition of MTOR.^99^ In line with this, branched‐chain amino acid supplementation leads to an inhibition of autophagy by activation of MTOR.^100^ Furthermore, overall low energy status, DNA damage, and hypoxia can inhibit MTOR through AMPK and hypoxia inducible factor 1, alpha subunit (HIF1‐α)^101^, ^102^ and thereby induce autophagy. Interestingly, exercise targets these factors, and exercise training results in elevated levels of basal autophagy.^103^ However, exercise leads to an increase in muscle mass and strength in COPD patients.^98^ Moreover, autophagy is required for muscle adaptations to training.^103^, ^104^ The counterintuitive effect of exercise on autophagy may therefore be more tightly linked to its function in quality control.

A more speculative thought is that autophagy may play a role in selective removal of damaged myonuclei. Besides causing mitochondrial DNA (mtDNA) damage, oxidative stress also causes nuclear DNA damage. COPD patients display elevated levels of oxidative stress, which may lead to increased DNA damage and requires increased removal of damaged nuclei. Although exercise induces oxidative stress, which is even accentuated in COPD patients,^105^ exercise also triggers myogenesis.^106^ In COPD, this myogenic response to exercise seems intact, although it is specifically impaired in cachectic COPD.^40^, ^98^ Exercise‐induced satellite cell activation is mediated by IGF signalling.^107^ In contrast, MSTN signalling inhibits satellite cell activation.^108^ Because MSTN expression is increased in COPD, pharmacological inhibition of MSTN might be beneficial to prevent COPD‐induced muscle wasting. This idea is supported by the finding that in mice with chronic kidney failure, pharmacological inhibition of MSTN blocks muscle atrophy,^109^ and that pharmacological inhibition of the ActII‐receptor, which mediates MSTN signalling, prevents glucocorticoid‐induced muscle atrophy.^110^ *In vitro*, glutamine reduced the tumour necrosis factor alpha‐dependent increase of MSTN.^111^ This implicates that availability of amino acids is important for normal satellite cell function in COPD and that restoration of normal amino acid levels may be required for muscle maintenance. Taken together, the dual function and differential regulation of UPS and autophagy in the maintenance of muscle mass and quality reflects a highly interactive signalling network that is regulated by several upstream pathways. The effect of specific modulation of UPS and autophagy mediators may therefore transcend catabolic signalling and may affect a range of other cellular processes, yielding it difficult to predict long‐term side effects. So far, exercise seems to be the only intervention that can target UPS and autophagy leading to improved quantity, as well as an improved quality of the muscle in COPD patients. One prerequisite is that COPD patients, and specifically cachectic COPD patients, have maintained responsiveness to exercise stimuli, which remains to be established. Exercise capacity in COPD may be limited by impaired pulmonary function, leading to incapability to supply a sufficiently strong exercise trigger to the muscles. In this case, pharmacological or nutritional activators of AMPK, sirtuin 1, and peroxisome proliferator‐activated receptors such as metformin, resveratrol, rosglitazone, and polyunsaturated fatty acids could be used as exercise mimetics and may help sensitize the muscle to a following exercise bout. Furthermore, anabolic steroids could be considered in the treatment of COPD‐induced muscle wasting, although a recent meta‐analysis showed that exercise capacity of COPD patients was not improved.^112^ It should also be considered that an appropriate nutritional status is necessary for the beneficial effects of exercise and that exercise (in particular, endurance type of exercise) in a malnourished state could even have detrimental effects by worsening the energy imbalance. Taken together, a multi‐modal approach may be required to combat COPD‐induced muscle wasting, in which exercise training is central. However, to establish such intervention, further research is crucial to determine whether the response to exercise is intact or if specific defects occur, in cachectic patients with COPD.

## Putative mechanisms involved in a disturbed energy balance in COPD

Specific loss of muscle mass in weight‐stable COPD patients has been observed, which may reflect a tissue‐specific sensitivity to an overall catabolic state. A net catabolic state may also result from an imbalance in energy expenditure and energy availability (energy balance).

### Increased energy expenditure

It is well established that components of whole body energy expenditure may be increased in patients with COPD.^113^ Total daily energy expenditure (TEE) is the sum of resting energy expenditure (REE), activity‐induced energy expenditure (AEE), and diet‐induced thermogenesis (DIT). Assessment of TEE requires sophisticated methodology including a respiration chamber^114^ or doubly labelled water to allow assessment of TEE in free living conditions.^115^, ^116^ Data on TEE in COPD are scarce and sometimes contradicting, which may be related to the use of different methodology or patient characteristics. Based on the available evidence, it seems that in particular the emphysematous phenotype is prone to increased TEE, as high values are more often observed in patients with low carbon monoxide diffusing capacity.^117^, ^118^, ^119^ This is in line with a positive effect of lung volume‐reduction surgery on body weight in emphysema, although surgical intervention might not only decrease energy requirements but also improve dietary intake by alleviating dyspnoea.^120^ More research is needed to assess putative differences in whole‐body energy metabolism and its components among COPD phenotypes under comparable standardized circumstances.

Numerous studies have shown that REE is raised.^121^, ^122^, ^123^ This is more prevalent in emphysema^124^, ^125^ during acute exacerbations,^126^ and appears inversely correlated with forced expiratory volume in 1 s when comparing different studies.^118^, ^119^, ^122^, ^127^ Highest values are found among weight‐losing patients.^122^ This is in contrast with non‐pathology‐induced malnutrition, where subjects with low BMI have lower REE due to hypometabolic adjustments.^128^ The same results are found for non‐small‐cell lung cancer (NSCLC), where REE is found to be unregulated in 74% of primary lung cancer patients.^129^, ^130^, ^131^ Hypermetabolism at rest was also found to be more pronounced in weight‐losing compared with weight‐stable lung cancer patients.^132^ Thus, increased REE is a consistent feature of chronic and more acute cachexia and seems to be more pronounced in the emphysematous subtype.

Activity‐induced energy expenditure is the most variable component of TEE, and it has been postulated that COPD patients reduce physical activity to compensate for dyspnoea severity or to anticipate to breathlessness. Indeed, lower physical activity levels are seen in COPD.^133^ Physical inactivity is associated with advanced disease stage,^134^, ^135^, ^136^ exacerbations,^137^, ^138^ and degree of emphysema.^139^ In addition, lung volume‐reduction surgery in patients with severe emphysema improved exercise performance due to reduced lung hyperinflation, less dyspnoea severity, and less cost of breathing.^140^ However, it did not cause augmentation of physical activity level, implying other factors play a role, including motivation or anxiety.^141^ There are several indications that when COPD patients perform physical activities, they require more energy. For example, Lahaije *et al*. found a higher daily activity‐related oxygen consumption assessed by a face mask measuring ventilatory and metabolic demand in COPD patients compared with healthy controls,^142^ while Vaes *et al*. found an increase in FFM adjusted oxygen consumption in COPDpatients compared with controls, although total oxygen consumption was not altered.^143^ Indirect evidence for altered AEE is a rise in plasma ammonia in COPD patients during low intensity walking, which is an indicator of muscle ATP depletion and metabolic stress.^144^ These collective data may indicate that COPD patients use oxygen less efficiently and exhibit an altered energy metabolism during physical activity. This is not surprising in view of the shift in lower limb muscle fibre type composition in COPD towards less oxidative fibres, which appears to be more pronounced in the emphysematous phenotype.^18^ The opposite shift in muscle fibre type of the diaphragm relative to the limb muscle^47^, ^48^ indicates an adaptation to chronic increase in work of breathing. Together with hyperinflation‐induced mechanical inefficiency, this muscle fibre type shift could contribute to enhanced oxygen cost of breathing, illustrated by the effects of lung volume‐reduction surgery^145^ and non‐invasive positive‐pressure ventilation therapy.^146^ For comparison, in lung cancer, physical activity level assessed by accelerometry is also reduced,^147^, ^148^ but no specific data about AEE.

Diet‐induced thermogenesis represents metabolic oxygen cost for processing of ingested nutrients. Green and colleagues^125^ indeed reported enhanced DIT in emphysematous COPD patients, but this was not confirmed by other authors,^149^, ^150^ independently of BMI.^151^ These differences may be due to different test meal composition and portion size. Although oxygen desaturation during meals was noticed in severe COPD patients,^152^ it is unknown whether this is DIT related or not. Therefore, the thermic effect of dietary intake remains unclear. Taken together, it indicates that energy requirements are increased in COPD, and there is certainly no adaptive reduced energy demand.

In addition to the hypermetabolic state, early clinical trials have shown that enhanced systemic inflammation is a contributing factor to elevated REE, both in COPD^153^ and in lung cancer,^154^ the source of which is yet unclear. Besides pulmonary inflammation,^155^ adipose tissue has also been suggested to contribute to a higher inflammatory gene expression in adipose tissue, as has been reported in malnourished patients with advanced COPD.^156^

### Adipose tissue metabolism

In cachexia, muscle wasting is accompanied by loss of adipose tissue.^14^, ^157^ In fact, in cancer‐induced cachexia, adipose tissue is often one of the first affected organs, illustrated by decreasing fat cell volume and upregulation of fatty acid metabolism.^158^ Regarding COPD, low BMI^11^ and fat mass depletion^14^ particularly occur in those with advanced disease and in the emphysematous phenotype.

Schols *et al*. observed low leptin levels in the blood of patients with emphysema compared with chronic bronchitis in line with a lower BMI and fat mass.^159^ After adjustment for FM and oral corticosteroid use as possible confounders, leptin was associated with systemic inflammation, in particular in the emphysematous patients. More recently, Brusik *et al*. investigated serum levels and adipose tissue expression of leptin and adiponectin in patients with COPD and reported an association between decreased serum and tissue leptin levels, and decreased serum adiponectin and increased REE adjusted for body weight in underweight patients.^160^ In adipose tissue, two cell types can be distinguished: white adipose tissue (WAT) and brown adipose tissue (BAT). Brown adipose tissue is differentiated from WAT by the presence of cold and diet‐induced thermogenesis. Thermogenesis is facilitated by BAT‐specific uncoupling proteins (UCP) that dissipate the proton gradient in mitochondria in order to generate heat.^161^ High amounts of mitochondria and high vascularisation are responsible for the brown colour of BAT.^162^ Additionally, WAT can be converted in BAT, called WAT browning.^163^ BAT activation negatively correlates with BMI, as demonstrated by decreased BAT activation in obese subjects^164^ and during ageing.^165^ Cachexia on the other hand is characterized by fat mass depletion. This raises the question whether there is a role of BAT activity in the hypermetabolic state, as seen in pulmonary cachexia.

No studies are performed to determine BAT activity in COPD patients. With respect to lung cancer, results are conflicting and scarce. Despite negative results of BAT activation reported by some authors,^166^, ^167^ Shellock and colleagues provided evidence for BAT activation as a cachexia mediator. Autopsy reports of cachectic cancer patients revealed high incidence of BAT in this group compared with age‐matched controls.^168^ Furthermore, a correlation between BAT activity and neoplastic status has been suggested,^169^ although the authors also reported high amount of BAT activation in non‐malignancy subjects.

There is indirect evidence that BAT activation might be a potential cachexia driver in COPD as well. Hypoxia^170^ and hypermetabolism^122^ are hallmarks of COPD. In response to hypoxia, cells can produce vascular endothelial growth factor (VEGF) in order to restore oxygen supply.^171^ This has been established by Van Den Borst *et al*., who found an upregulation of the VEGF gene in adipose tissue in response to chronic hypoxia in mice. Congruently, adipose tissue showed a brown appearance. This browning of adipose tissue was established by increased expression of UCP1,^172^ which proposes a link between hypoxia‐induced VEGF activation and browning. Indeed, Sun *et al*. revealed upregulation of UCP‐1, the main characteristic of BAT, in a VEGF overexpressing mouse model.^173^ In addition, recently, increased thermogenesis and energy expenditure were found in mice with VEGF overexpression in BAT and WAT.^174^

Another hypoxia alignment occurs in the form of lactate. In peripheral muscle of COPD patients, increased glycolysis metabolism is observed,^175^ which in turn causes rising lactate levels.^176^, ^177^ Lactate is indeed increasingly released by adipocytes in a hypoxic environment,^178^ which in turn is able to control the expression of UCP‐1. The UCP‐1 regulation is independent of HIF‐1α and thereby also promotes WAT browning under normoxic circumstances.^179^

Another possible browning factor is beta‐adrenergic stimulation mediated by norepinephrine.^180^, ^181^ Emphysematous COPD patients indeed exhibit increased plasma norepinephrine levels,^182^ indicating a possible activation of the autonomic nervous system. However, Schiffelers *et al*. showed a blunted beta‐adrenoceptor‐mediated lipolytic and thermogenic response,^183^ suggesting desensitization. Additionally, in 10 lean healthy men, BAT activity in response to a systemic non‐selective beta‐agonist was not enhanced.^184^ In contrast, blocking the receptors by propranolol decreases BAT activity.^185^ Therefore, activation of brown fat through beta‐adrenergic stimulation remains disputable.

It can be concluded that there is some indirect evidence pointing towards a role of BAT in pulmonary cachexia, but this area requires more research to identify therapeutic potential.

## Compromised dietary intake

In order to compensate for increased energy requirements in COPD, patients should be able to adapt their dietary intake. Systematic analyses of dietary intake in COPD patients are rare. In terms of caloric content, dietary intake was found to be normal compared with healthy controls, but inadequate for measured energy expenditure.^118^, ^186^, ^187^, ^188^ During severe acute exacerbations, the gap between energy intake and energy expenditure becomes even wider, which slowly decreases upon recovery.^126^, ^189^ To our knowledge, no human studies have systematically investigated the relation between dietary intake and disease severity or putative differences between emphysematous and non‐emphysematous patients. Advanced disease stages and acute exacerbations are often characterized by chronic or acute hypoxemia.^170^ It is well established that mice under chronic hypoxic circumstances experience weight loss, which is partly due to temporarily decreased dietary intake.^172^

### Anorexia

It is acknowledged that apparently normal dietary intake in COPD patients may be insufficient to meet elevated energy requirements, but reduced food intake may also be caused by anorexia, that is, loss of appetite.^190^ A few underlying causes have been mentioned, including nicotine use,^191^ physical discomfort such as dyspnoea and increased breathing effort,^192^ depression, and anxiety,^193^ seen in COPD^194^ as well as in NSCLC.^195^, ^196^

Besides pulmonary and psychological symptoms, COPD patients often experience pain. In a Norwegian study, which controlled for age and gender, 45% of the COPD patients reported chronic pain, compared with 34% of the general population.^197^ Opioids are commonly used to combat pain in COPD.^198^ Side‐effects of opioids occur regularly, and opioids are able to cause gastrointestinal motility disorders,^199^ of which constipation is the most common.^200^ People suffering from constipation often present with anorexia,^201^ probably due to early satiety. Separate from use of pain medication, early satiety and abdominal bloating is highly prevalent in COPD.^202^

### Chemosensory alterations

Food intake is regulated by taste and smell,^203^, ^204^ and chemosensory dysfunction could influence dietary intake. Nordén *et al*. showed that 21 out of 169 stable COPD patients reported taste changes, which contributed to a decreased energy intake.^205^ In addition, Dewan *et al*. compared 20 COPD subjects with long‐term oxygen therapy to 20 COPD patients without oxygen therapy and 20 healthy elderly controls. They found reduced smell and taste test scores among COPD patients compared with controls, independent of oxygen supply.^206^ Also, Wardwell and colleagues found that healthy elderly tended to be able to identify more different tastes correctly than COPD patients, although not statistically significant.^207^ Both authors did not report medication use, and therefore, the influence of treatment is unknown. Although data are scarce and methodological quality of the studies is limited, these data suggest that COPD or its treatment could modify taste and smell detection.

### Food reward system

Fullness is regulated by gastrointestinal hormones, including leptin and ghrelin,^208^ and their secretion is affected by dietary intake and nutritional status. Clinically stable emphysematous COPD patients exhibit low leptin levels compared with the chronic bronchitis subtype.^159^ During acute flare ups, these plasma levels rise temporarily,^189^ as seen in NSCLC.^209^, ^210^ Likewise, enhanced plasma ghrelin levels are noticed in COPD^211^ and NSCLC^212^ and are related to cachectic status.

The peripheral hormonal satiety system closely interacts with the central nervous system in order to regulate food intake. Brain imaging studies have revealed reward‐specific brain regions related to food reward,^213^ and activation of these regions correlate with food rewarding.^214^ Different orexigenic and anorexigenic peptides and hormones can stimulate neurons in these specific cerebral regions.^208^, ^215^ For instance, leptin inhibits neurons, causing reduced food intake and increased energy expenditure.^216^ Ghrelin, considered to be a leptin counterpart, can induce food intake mediated by stimulation of neurons in this area.^217^

There is surprisingly no human study available that explored the role of central dysregulation in food reward in patients with COPD. In relation to lung cancer cachexia, only one study was performed identifying brain activity in anorectic and non‐anorectic patients while receiving pleasant and unpleasant food cues.^218^ In contrast to non‐anorectic patients, anorectic patients showed no brain activity differences in response to pleasant versus unpleasant pictures. This implies an overall blunted response in the perceptual and motivational system that could also be involved in COPD but requires further investigation.

### Therapeutic perspective

The importance of nutritional status is not only emphasized by adverse effects on muscle function and exercise performance but also by detrimental effects of malnutrition on lung tissue. These effects have mostly been studied in animal models. Following the clinical phenotyping of the pink puffer and the blue bloater in the 1960s, Sahebjami *et al*. found reinforcement of pre‐existing emphysematous processes due to caloric food deprivation in rats,^219^ which was more pronounced in young rats.^220^ These deleterious effects could partly be reversed by refeeding.^221^ In contrast, Bishai *et al*. found no alveolar size changes in calorie‐restricted mice, although the lungs became stiffer and lung capacity was decreased.^222^ Supplementary evidence was provided by emphysema‐like changes present in anorexia nervosa patients, which underscores the impact of chronic malnutrition on alveoli.^223^ In addition to lung tissue, respiratory muscles also contribute to breathing. Weight loss does not spare the respiratory muscles, because weight loss is related to diminished diaphragm weight^224^ and decreased function^225^ in experimental models and in humans.

As proof of concept, Efthimiou *et al*. conducted a randomized controlled trial to investigate the effect of nutritional support on respiratory and peripheral muscle function in malnourished COPD patients. They reported improvement in respiratory muscle strength and hand grip strength, accompanied by less dyspnoea and enhanced distance in 6‐min walk test. Importantly, these effects diminished after quitting the dietary supplementation.^226^ The positive effects of dietary support on body weight was verified by Weekes *et al*., who found weight gain in the intervention group, whereas the control group continued to lose weight. Addition of dietary counselling to dietary support has been shown to maintain weight loss after cessation of intervention.^227^

Initially, the focus was primarily on caloric intake to balance energy requirements, but more recent proof of concept experiments also highlighted the importance of optimizing protein intake.^228^, ^229^ Low intake of other essential nutrients is identified, including vitamin D and calcium,^230^ which are also relevant in the context of osteoporosis as clustering comorbid condition.

One should keep in mind that dietary intake does not reflect actual availability of ingested micronutrients. There are indications that intestinal function is impaired in COPD, illustrated by splanchnic hypoperfusion and reduced intestinal permeability.^231^ Altered intestinal function translates into reduced splanchnic extraction of amino acids derived from nutritional intake,^36^, ^232^ but as a result, the amino acid uptake in the skeletal muscle of clinically stable COPD patients appears increased.^233^ Thus, the significance for clinical applications remains ambiguous.

Both dietary intake and nutrient availability are controlled by gastrointestinal hormones. By binding to the growth hormone secretagogue receptor, ghrelin can induce secretion of growth hormone.^234^ This leads to modulation of the growth hormone/IGF1 axis, which is an important anabolic pathway in human skeletal muscle.^235^ Furthermore, ghrelin can induce food intake, mediated by stimulation of specific neurons in the food reward centre.^217^ Due to its orexigenic property, ghrelin analogues have been proposed for clinical application in cachexia. One clinical trial with ghrelin analogues have been conducted in COPD patients. They reported improvements of ventilatory efficiency at peak exercise, reflected by increased peak oxygen uptake.^236^ However, it did not translate in improved 6‐min walk distance, and no data are available about body composition or food intake. Clinical trials in cancer cachexia,^237^ including lung cancer,^238^ demonstrate an enhanced lean body mass and quality of life. Hence, ghrelin analogues warrant further investigation in COPD. Besides dietary and pharmacological interventions, cognitive behavioural interventions are relatively underexplored in the management of cachexia in COPD. Although results from different functional neuroimaging studies are inconsistent and sometimes conflicting,^239^ there might be altered reactivity in the brain reward system in response to perceived food stimuli in people with altered eating patterns, including anorexia nervosa and obesity.^240^, ^241^ Therefore, cognitive behavioural therapy may serve as a treatment for patients with an eating disorder like anorexia nervosa.^242^ Recently, a randomized controlled trial was conducted in obese subjects, receiving behavioural therapy for 6 months in order to reduce weight. Analysis of functional magnetic resonance imaging revealed changes in reward system activity in the intervention group versus controls. Further research has to identify whether it is possible to enhance neuroplasticity in the food reward centre in order to increase successfulness for eating disorder treatment.^243^ This opens up new insights and therapeutic opportunities for suspected nutritional therapy‐resistant cachectic COPD patients, if disturbances in the central food reward system are indeed identified.

## Conclusions

**Figure 1:**
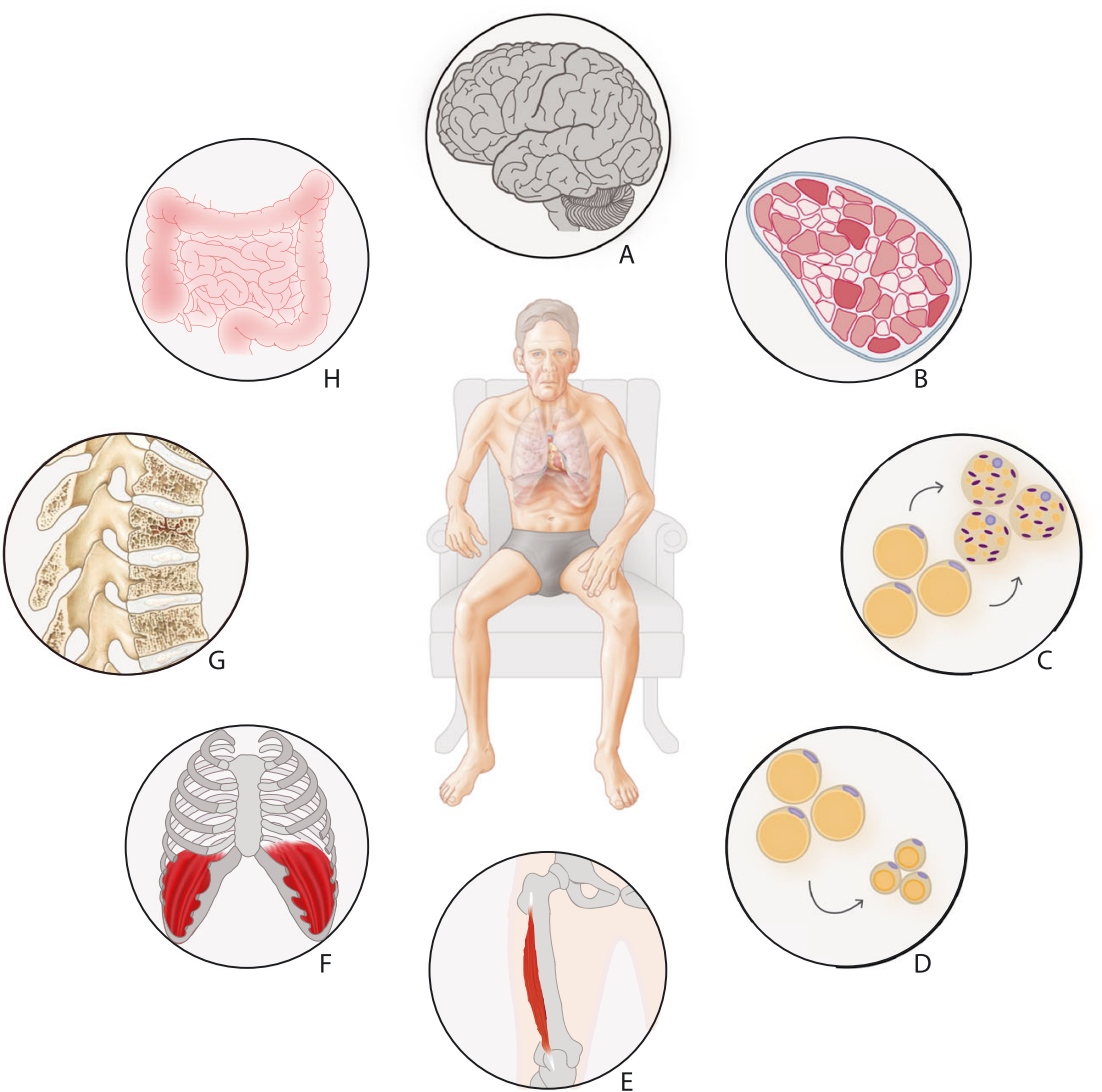
Pulmonary and extra‐pulmonary cross‐talk in COPD cachexia. (A) Altered brain responses to food stimuli; (B) muscle fibre type shifting and oxidative metabolism; (C) altered adipose tissue metabolism; (D) adipose tissue wasting; (E) limb muscle dysfunction; (F) respiratory muscle dysfunction; (G) osteoporosis; (H) altered gut integrity and reduced splanchnic extraction.

It is well established that the prevalence and related disease burden of cachexia is high in COPD and likely to increase in the near future given the high and increasing prevalence of the disease in an ageing population. Nevertheless, cachexia management is still poorly implemented in clinical practice. In 2014, the European Respiratory Society published a statement on nutritional assessment and therapy in COPD including a nutritional risk stratification diagram based on assessment of BMI, weight changes, and body composition, which could be useful in patient counselling.^24^ In order to increase overall survival and compress morbidity, a multi‐modal intervention approach is needed, which should target the discussed factors involved in cachexia (Figure 1). Such a multi‐modal intervention approach, encompassing exercise training and improvement of energy balance and nutrient availability, is currently feasible as supported by recent statements and meta‐analyses, possibly improved in the near future by targeted pharmacological interventions and cognitive behavioural therapy to sensitize patients to anabolic stimuli.

## Conflict of interest

None.
